# Microparticle conferred microRNA profiles - implications in the transfer and dominance of cancer traits

**DOI:** 10.1186/1476-4598-11-37

**Published:** 2012-06-08

**Authors:** Ritu Jaiswal, Frederick Luk, Joyce Gong, Jean-Marie Mathys, Georges Emile Raymond Grau, Mary Bebawy

**Affiliations:** 1 School of Pharmacy, Graduate School of Health Level 13, Building 1, University of Technology, Sydney, 123 Broadway, NSW, 2007, Australia; 2 Vascular Immunology Unit, Sydney Medical School and Bosch Institute, The University of Sydney, Sydney, NSW, 2006, Australia; 3 Department of Medicine, University of Connecticut Health Center Farmington, CT, 06032, Connecticut, USA

**Keywords:** Cancer, Microarray, Microparticles, MicroRNA, Multidrug resistance, Selective packaging

## Abstract

****Background**:**

Microparticles (MPs) are membrane vesicles which are released from normal and malignant cells following a process of budding and detachment from donor cells. MPs contain surface antigens, proteins and genetic material and serve as vectors of intercellular communication. MPs comprise the major source of systemic RNA including microRNA (miRNA), the aberrant expression of which appears to be associated with stage, progression and spread of many cancers. Our previous study showed that MPs carry both transcripts and miRNAs associated with the acquisition of multidrug resistance in cancer.

****Results**:**

Herein, we expand on our previous finding and demonstrate that MPs carry the transcripts of the membrane vesiculation machinery (*floppase* and *scramblase*) as well as nucleic acids encoding the enzymes essential for microRNA biogenesis (*Drosha, Dicer* and *Argonaute*). We also demonstrate using microarray miRNA profiling analysis, the selective packaging of miRNAs (miR-1228*, *miR-1246*, *miR-1308*, miR-149*, *miR-455-3p*, *miR-638* and *miR-923)* within the MP cargo upon release from the donor cells.

****Conclusions**:**

These miRNAs are present in both haematological and non-haematological cancer cells and are involved in pathways implicated in cancer pathogenesis, membrane vesiculation and cascades regulated by ABC transporters. Our recent findings reinforce our earlier reports that MP transfer ‘re-templates’ recipient cells so as to reflect donor cell traits. We now demonstrate that this process is likely to occur via a process of selective packaging of nucleic acid species, including regulatory nucleic acids upon MP vesiculation. These findings have significant implications in understanding the cellular basis governing the intercellular acquisition and dominance of deleterious traits in cancers.

## **Background**

Extracellular membrane vesicles are important vehicles of intercellular communication across numerous biological processes. MPs are typically defined by their size (0.1-1 μm in diameter) [1], exposure of phosphatidylserine (PS) and the expression of surface antigens originating from their donor cells [1-3].

MP vesiculation occurs as a cellular response to various physiological conditions including; apoptosis, senescence, cellular activation [4]; shearing stress and biochemical triggers (such as cytokines and chemotherapeutics) [5]. In the steady state the cell membrane is asymmetric in its composition with phosphatidylcholine and sphingomyelin located in the outer layer whereas phophatidylserine (PS) and phosphatidylethanolamine (PE) present in the inner layer. This asymmetric distribution in the membrane is maintained by a group of two ATP-dependent enzymes namely *flippase, floppase* as well as a bidirectional ATP-independent *scramblase*[6-8]. Flippase specially translocates PS and PE from the outside to the inside of the bilayer membrane. Floppase transports phospholipids and cholesterol from the inner to the outer leaflet. Floppase does not specifically act on transport of aminophospholipids and probably works together with flippase. Scramblase whose role is thought to be the transportation of phospholipids between the two monolayers of the cell membrane, is inactive in steady state [6-8]. Following stress or under physiological conditions, an increase in intracellular calcium, a subsequent loss of phospholipid asymmetry following the inactivation of *flippase* and activation of *floppase* and *scramblase*, and disruption of the cytoskeletal apparatus occurs leading to MP vesiculation [6,7,9]. The released MPs are enriched in PS and PE exposed on their outer surface. Consequently, MPs carry also cellular proteins, second messengers, growth factors and genetic material from their cells of origin [1,10] and comprise the major source of RNA (ribosomal RNA (rRNA), messenger RNA, (mRNA) and microRNA (miRNA) in systemic circulation [11,12].

miRNAs are highly conserved, single-stranded non-coding regulatory nucleic acids, typically 19–25 nucleotides in length. These RNAs modulate the activity of specific mRNA targets and serve as important regulators of a wide range of pathophysiological processes [13]. miRNA synthesis begins in the nucleus by RNA polymerase II to form primary miRNA (pri-miRNA). Pri-miRNA is processed by the ribonucleases, *Drosha* and *Dicer* to generate mature miRNA. The single stranded miRNA, in association with *Argonaute 2*, binds to complementary sequences in the 3' untranslated region (UTR) of target transcripts to regulate gene expression either by translational repression, activation or degradation of the mRNA transcript [1,14]. By targeting several genes, miRNAs play important roles in normal biological processes including cell proliferation, differentiation, apoptotic cell death, stress resistance and physiological metabolism [15,16]. Consequently, aberrant expression of miRNAs has been associated with malignancy, including; cancer stage, disease progression and metastasic spread [17-19]. Furthermore, some miRNAs have been shown to have oncogenic (such as *mir-21*, the cluster *mir-17–92*, *miR-155*, *miR-221* and *miR-222*) [20] and tumour suppressive (such as let-7 in lung cancer and *miR-15/16* in leukaemia and prostate cancer) properties [21-23].

Given that MPs are emerging as an important source of miRNA in the circulation in cancer patients [24-26] it is feasible to propose a role for MP in the aberrant miRNA levels displayed in oncogenesis and spread. This reinforces the role that MPs play in cancer biology including cell survival, invasion, metastasis and angiogenesis [27-31]. We recently discovered that MPs serve an important function as mediators in the dissemination and acquisition of multidrug resistance in cancer [32]. Specifically, we have demonstrated that this occurs via the MP-mediated transfer of functional resistance proteins, and nucleic acids including regulatory nucleic acids. In addition, we also showed that the MP transfer ensured the acquisition of the donor cell trait on to the recipient cells [33].

We now expand on these findings and demonstrate that MPs carry the transcripts encoding the membrane vesiculation machinery (*floppase* and *scramblase*) and the enzymes essential for microRNA biogenesis (*Drosha, Dicer* and *Argonaute*). We also demonstrate the selective packaging of miRNAs within MP cargo upon release from the donor cells and propose that this process contributes to the dissemination and acquisition of the donor cell trait.

## **Results**

### **Microparticles incorporate transcripts encoding the vesiculation machinery and microRNA biogenesis enzymes**

**Figure 1:**
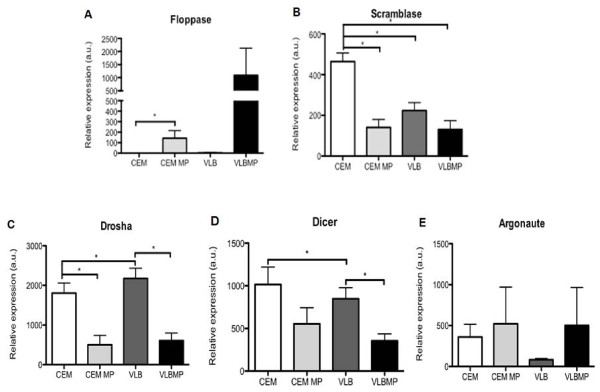
**MPs incorporate transcripts of vesiculation machinery and microRNA biogenesis enzymes.** Quantitative RT-PCR showing the levels of transcripts of vesiculation machinery (**A**) *Floppase* and (**B**) *Scramblase* and microRNA biogenesis enzymes (**C**) *Drosha* (**D**) *Dicer* and (**E**) *Argonaute 2* transcripts in CEM cells (white), CEMMP (light gray), VLB_100_ cells (dark gray) and VLBMP (black). Values are expressed as relative expression with respect to the endogenous control gene, GAPDH. Data represent the mean ± SEM of 3 independent experiments conducted in duplicate *p < 0.05

qRT-PCR analysis of leukaemic cells and their MPs show that both the drug sensitive and resistant parental cells as well as their MPs, carry the transcripts for the vesiculation enzymes, *floppase* and *scramblase* (Figure 1A and 1B). *Floppase* is present at significantly higher levels in the MPs relative to the donor cells (Figure 1A), whereas *scramblase,* though present in all samples is present at significantly lower levels in the resistant cells and their MPs relative to the sensitive parental cells (Figure 1B).

MPs originating from VLB_100_ and CEM cells carry the transcripts encoding the enzymes *Drosha*, *Dicer* and *Argonaute* (Figure 1C, D and E), required for miRNA biogenesis. Both the drug sensitive and the resistant cells have significantly higher levels of the transcripts for *Drosha* and *Dicer* relative to their MPs (Figure 1C and D). *Argonaute* is also present in both the cells and their MPs but with no significant differences in their levels (Figure 1E).

### **Presence of miRNAs and modulation of the recipient cell miRNA profile following microparticle transfer**

**Figure 2:**
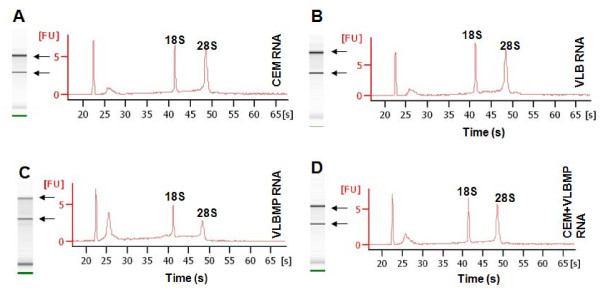
**RNA integrity of samples.** RNA derived from (**A**) the drug sensitive-recipient cell (CEM), (**B**) drug-resistant VLB_100_ cells, (**C**) their isolated MPs (VLBMP) and (**D**) the drug sensitive-recipient cells after MP transfer (CEM + VLBMP) was analysed using Agilent RNA 6000 Nano kit by Agilent 2100 Bioanalyzer. The RIN value of the samples ranged between 6.2-9.2. Data is representative of a typical experiment

**Figure 3:**
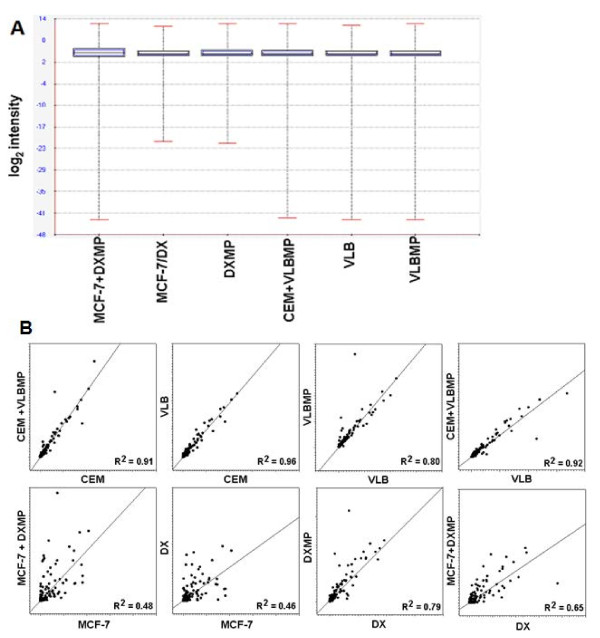
**Quality of data and gene expression signal correlation among identified miRNA.** After normalization and transformation of the microarray data (**A**) the box-whisker plot of probes signal intensity assessed and confirmed the microarray data quality. (**B**) The scatter plot of the signal intensity of the 847 annotated human miRNAs showed that certain level of correlation was identified between the MPs (VLBMP or DXMP), the acquired cells (CEM + VLBMP or MCF-7+ DXMP) , the donor cells (VLB_100_ or DX) and the parental recipient cells (CEM and MCF-7)

**Figure 4:**
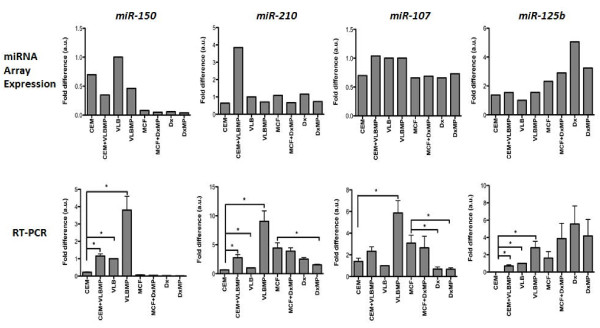
**Validation of microarray data by qRT-PCR.** By using qRT-PCR, *miR-150***,***miR-210, miR-107* and *miR-125b* were analyzed in MPs, their donor cells, the recipient cells before and after MP transfer across both leukaemia and breast cancer. Results depict similar trends in gene expressions across samples by both qRT-PCR and microarray. qRT-PCR expression levels were normalized with respect to the endogenous control gene, U6 whereas the microarray expression levels were normalized with respect to the human 5.8 s rRNA (gi555853). Data expressed as fold differences represents the mean ± SEM o f 2 independent experiments conducted in triplicates * *p* < 0.05

The quality of isolated RNAs was confirmed before subjecting the samples to miRNA microarray analysis (Figure 2). After normalization and transformation of the microarray data, the box-whisker plot of probe signal intensity was used to assess and confirm the quality of the microarray data (Figure 3A). Among the 7,815 probe sets in the miRNA microarray (http://www.affymetrix.com/support/technical/datasheets/miRNA_d atasheet.pdf), 847 probes were annotated as human miRNAs. The scatter plot of the signal intensities of these 847 human miRNAs displayed a correlation between MPs and their donor cells as well as between the acquired cells and the donor cells (Figure 3B). The miRNA microarray data was validated by qRT-PCR using the following selected miRNAs namely *miR-150, miR-210, miR-107* and *miR-125b* (Figure 4).

**Figure 5:**
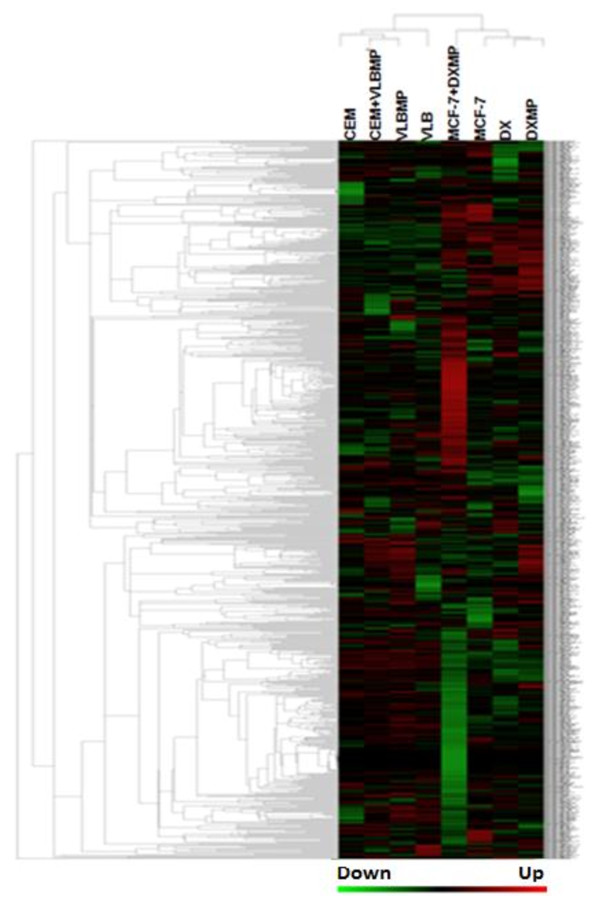
**miRNA expression profiles of MPs, their donor drug resistant cells, the recipient drug sensitive cells and the cocultures after MP transfer.** Hierarchical clustering analysis of the 847 human miRNA signal intensities identified by the Affymetric miRNA microarray detectable in the drug resistant MPs (VLBMP and DXMP), their donor cells (VLB_100_ and DX) and the drug sensitive recipient cells before (CEM and MCF-7) and after MP transfer (CEM + VLBMP and MCF-7 + DXMP) across both leukaemia and breast cancer samples. This shows evidence of the tight correlation between the MPs, cocultured cells and MP donor cells. Vertical bars represent the samples and the horizontal bars represent the miRNA genes. Green bars reflect downregulated genes and red bars upregulated genes

To explore those miRNAs that were involved in the transfer of drug resistance by MPs to recipient cells, the miRNA expression profiles of MPs, drug sensitive recipient cells, acquired cells and donor cells were compared. The hierarchical clustering analysis of the 847 human miRNA uncovered selectively packaged miRNAs in the MPs relative to the donor cells (Figure 5). Furthermore, the acquired cells displayed a miRNA profile consistent with the donor following MP transfer. The sensitive cells were differential in their miRNA expression with respect to their drug resistant counterparts. In total, 209 miRNAs in leukaemia and 215 in the breast cancer cells were differentially expressed between the resistant donor cells and their MPs (Figure 5). Also, 222 and 155 miRNAs were differentially expressed between the acquired cells following MP transfer and the donor cells, in leukaemia and breast cancer, respectively (Figure 5). 208 and 200 miRNAs were also found to be differentially expressed between the drug sensitive and the resistant cells, in leukaemia and breast cancer, respectively (Figure 5). Of these, 195 miRNAs in leukaemia and 140 miRNAs in breast cancer were commonly identified between these two comparisons. The high level of similarity indicated the strong relationship between the MPs, the acquired cells and the donor cells. In addition, hierarchical clustering analysis of the 847 human miRNA expression profiles between all samples displayed common trends across the two cancer cell lines and provides further evidence of the tight correlation between the MPs, acquired cells following MP transfer and the donor cells (Figure 5).

**Figure 6:**
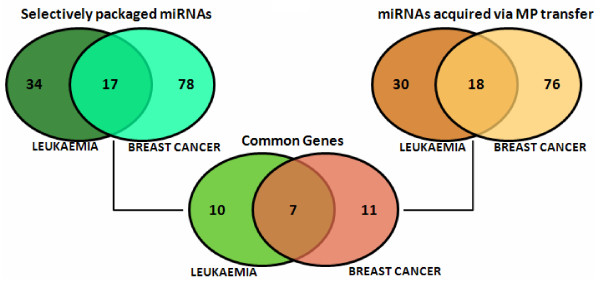
**Identification of significantly expressed miRNA by comparison profiles.** The Venn diagrams depicts 51 (leukaemia) and 78 (breast cancer) significantly and differentially expressed miRNA genes that were selectively packaged, whereas 48 (leukaemia) and 94 (breast cancer) significantly and differentially expressed miRNA that were acquired via MP transfer. Finally, 7 of the miRNA genes were co-detected across both cancers that were selectively packaged in the MP and were acquired by the recipient cells, after MP transfer. Significant miRNA genes having p < 0.06 were selected for analysis and from which those having fold change > 1.5 were identified as differentially expressed across both cancers

**Figure 7:**
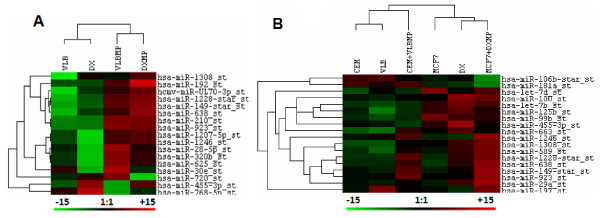
**MPs selectively package miRNA genes.** Hierarchical clustering analysis of leukaemia and breast cancer (**A**) resistant cells (VLB_100_ or DX) versus their MPs (VLBMP or DXMP) show clear differences in the expression of genes between the cells and the MPs depicting some miRNAs are selectively packaged into MPs. (**B**) Recipient cells resemble the donor after MP co-incubation. Hierarchical clustering analysis of leukaemia and breast cancer sensitive recipient cells (CEM or MCF-7), resistant donor cells (VLB_100_ or DX) versus the recipient cells cocultured with MPs (CEM + VLBMP or MCF-7 + DXMP) shows that the recipient miRNA gene expression trends follow that of the donor cells after MP co-incubation across both cells types. Heatmaps show the signal intensities of the co-detected miRNAs which have higher expression levels in the MPs whereas similar expression levels in the coculture with respect to its donor cells. The identified miRNAs have a fold change > 1.5 and *p-*value < 0.06. Green bars reflect downregulated genes and red bars upregulated genes

To identify the most prominent miRNAs, linked with the MP-mediated transfer of drug resistance trait to drug sensitive cancer cells, selectively packaged and acquired miRNAs having *p*-value less than 0.06 (*p* < 0.06) and fold change more than 1.5 (FC > 1.5) were selected (Figure 6). This comparison between leukaemia and breast cancer cells showed that 17 miRNAs were identified as the important miRNAs selectively packaged into MPs (Figure 7A). Likewise, across both cancers, 18 miRNAs were identified as significantly expressed miRNAs in the acquired cells (Figure 7B).

The MPs have a higher expression of these identified miRNAs relative to its donor cells, thereby being selectively packaged (Figure 7A), which are then transferred to the recipient cells upon coculture (Figure 7B). The acquired recipient cells display higher levels of miRNAs, relative to their parental recipient cells (CEM and MCF-7), following MP transfer. The expression levels in the MP acquired recipient cells with respect to their parental cells, is reflective of the donor cell trends (Figure 7B). Finally, 7 common miRNAs were identified as the most essential (being codetected across both malignancies), which may be important for the transfer of donor traits via MPs (Figure 6). The 7 miRNAs, which were identified to be selectively packaged and acquired by recipient cells across both cancers, include miR-1228*, *miR-1246*, *miR-1308*, miR-149*, *miR-455-3p, miR-638* and *miR-923* (Table 1).

**Table 1:** The list of identified miRNAs with their characteristics

**miRNA name**	**miRBase sanger accession number**	**Sequence**	**Length**	**Precursor sanger annotations**	**Chromosomal location**
hsa-miR-149-star	MIMAT0004609	5' - agggagggacgggggcugugc - 3'	21	MI0000478	2q37.3
hsa-miR-455-3p	MIMAT0004784	5' - gcaguccaugggcauauacac - 3'	21	MI0003513	9q32
hsa-miR-638	MIMAT0003308	5' - agggaucgcgggcggguggcggccu - 3'	25	MI0003653	19p13.2
hsa-miR-923	MIMAT0004973	5' - gucagcggaggaaaagaaacu - 3'	23	MI0005715	Fragment of the 28 S rRNA
hsa-miR-1228-star	MIMAT0005583	5’- ucacaccugccucgcccccc -3’	20	MI0006318	12
hsa-miR-1246	MIMAT0005898	5’- aauggauuuuuggagcagg -3’	19	MI0006381	2q31.1
hsa-miR-1308	MIMAT0005947	5’- gcaugggugguucagugg -3’	18	MI0006441	Fragment of a tRNA

### **Microparticles selectively package miRNAs implicating traits specific to membrane vesiculation, cancer etiology and multidrug resistance on the target cell**

**Figure 8:**
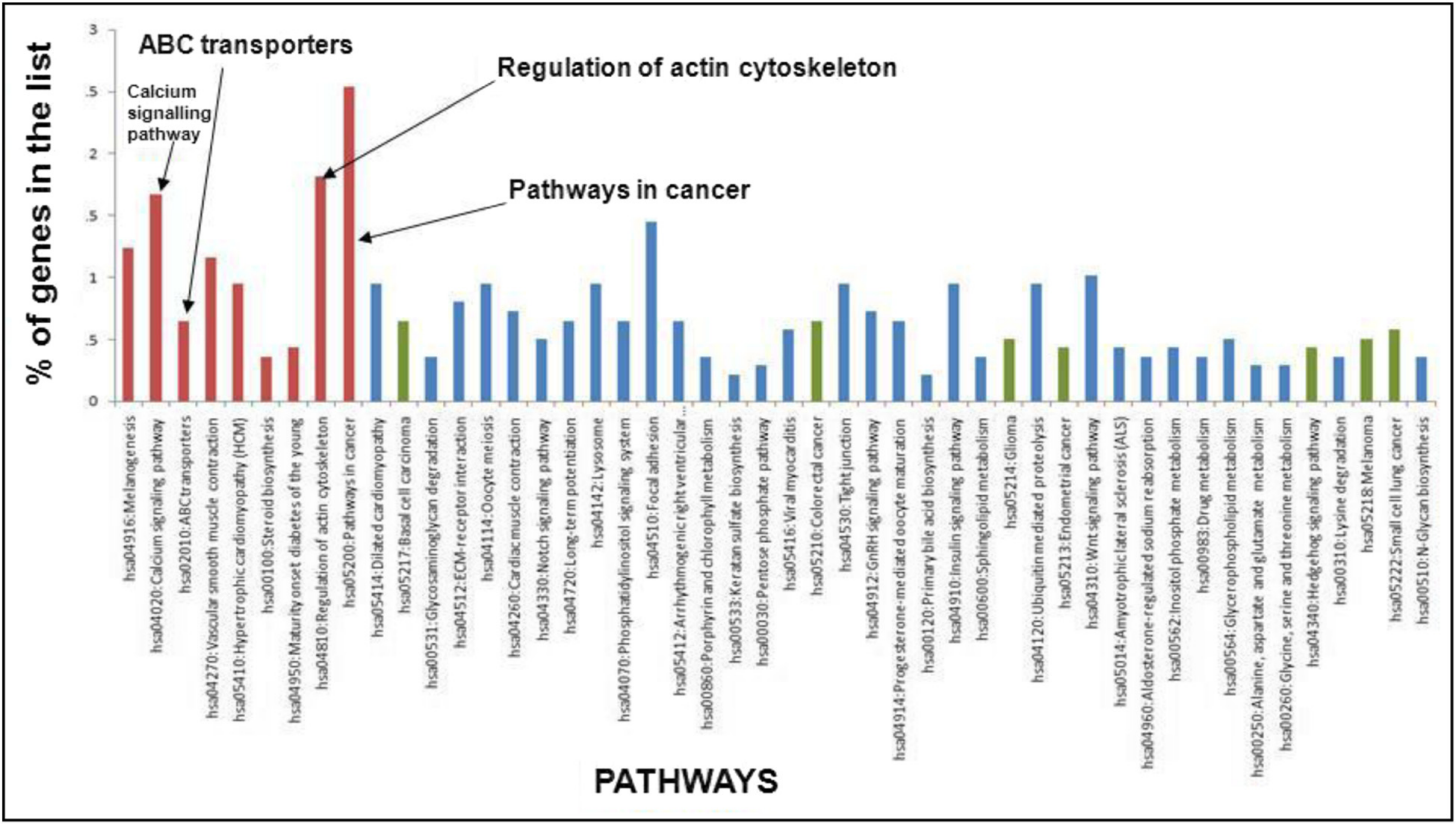
**Selected miRNAs have common biological pathways.** The common biological pathways were identified with the list of predicted targets for the seven co-detected miRNA genes by DAVID Bioinformatics Resources. The percentages of the total identified target genes (y-axis) were plotted against the pathways they regulate (x-axis) with increasing *p-*value. The pathways in red bars indicate most significant pathways with *p-*value < 0.05. The green bars indicate the pathways associated with malignancies. Significant biological pathways (*EASE score < 0.05) were selected as the important pathways. *Ease Score Threshold (Maximum Probability): The threshold of EASE Score, a modified Fisher Exact *p*-value, for gene-enrichment analysis. *p*-value <0.05 represents strong enrichment in the annotation categories

The 1,571 unique predicted gene targets of the 7 miRNAs were identified from miRBase (prediction score > 60) and EMBL (*p* < 0.01). The gene target list was uploaded to DAVID Bioinformatics Resources 6.7 web-based program for functional annotation analysis. Significant biological pathways (*EASE score < 0.05) were selected as the important pathways that may be involved in MP formation and MDR trait transfer to recipient cells. The top 9 significant correlated pathways (*p* < 0.05) include “melanogenesis”, “calcium signalling pathway”, “ABC transporters”, “vascular smooth muscle contraction”, “hypertrophic cardiomyopathy” “steroid biosynthesis”, “maturity onset diabetes of the young”, “regulation of actin cytoskeleton” and “pathways in cancer” (Figure 8). Of these significant pathways identified for the miRNAs in this study, two were related to MP vesiculation (“**calcium signalling pathway**”, and “**regulation of actin cytoskeleton**”) and one to MDR (“**ABC transporters**”). Of all the target genes identified for the miRNAs, the highest percentages (~2.5 %) of these were observed to be related to the “**pathways in cancer**”. In addition, of all the predicted pathways identified for the miRNAs in this study, eight of them were related to malignancies alone.

## **Discussion**

This study demonstrates that MPs serve as vehicles for intercellular communication and potentially as cancer biomarkers through their discrete miRNA signatures. RT-PCR analysis showed that MPs carry the transcripts of their vesiculation machinery (*floppase* and *scramblase*) (Figure 1A and B) together with transcripts encoding miRNA biogenesis enzymes (*Drosha, Dicer* and *Argonaute*) (Figure 1C, D and E). The presence of these phospholipid enzymes (*floppase and scramblase)* may help in intracellular vesicle trafficking either by inducing membrane vesiculation or by providing an environment favorable for binding of vesicle coat proteins [34]. This finding suggests that MPs are self-sufficient and possess the capacity to potentially induce vesiculation in the recipient cell. Although, the presence of scramblase 1 and 3 in exosomes has been previously reported [20,35], to our knowledge, this is the first demonstration of the presence of transcripts encoding the vesiculation machinery in MPs. The presence of *Drosha*, *Dicer* and *Argonaute* transcripts within the shed cargo potentially implicates MPs as key intercellular regulators of miRNA biogenesis in recipient cancer cell populations.

Affymetrix miRNA microarray was used to explore the miRNA expression profiles of MPs and their donor cells from both leukaemia and breast cancer cells in this study (Figure 5). This data was validated using RT-PCR studies where we observed almost similar trends for most miRNAs examined (*miR-107*, *miR-125b* and *miR-210*) except for *miR-150* (Figure 4). The basis of this difference in currently unknown and may be attributed to the differences in assays. Our analysis showed that several human miRNAs are selectively packaged into MPs. Upon coculture with recipient cells, we observe an increase in select miRNAs, inlcuding miR-1246, miR-1308, miR-1228*, miR-149*, miR-638 and *miR-923* (Figure 7).These miRNAs displayed a 2-12-fold increase in expression levels in the MPs relative to their donor cells (Figure 7A). These observations are consistent with selective packaging, which we have previously shown for *miR-451* and *miR-326* in MPs shed from MDR resistant breast and leukaemia cells [33]. In addition, our findings are consistent with previous reports demonstrating that these same miRNAs are also selectively packaged into exosomes [24,36-38].

We also demonstrate that following MP transfer to recipient breast cancer and leukaemia cells, the same miRNAs were significantly increased in the acquired cells, with levels ranging from 2-15-fold increase (Figure 7B)*.* Once again the expression level of the miRNAs in the MP compartment was directly correlated to the levels observed in the acquired population following MP transfer (Figure 5). In saying this however, we cannot exclude the possibility that increased miRNA levels in the recipient cells are caused by either/both direct or indirect MP-mediated effects on the transcription of the miRNA. Interestingly, the acquired population displayed miRNA expression trends reflective of the donor cells (Figure 7B). This demonstrates that the recipient cell reflects the donor trait after MP-mediated transfer of cargo. These findings are consistent with our previous report where we have shown the “**re-templating***”* of recipient cells to reflect donor cell traits following MP-mediated transfer of MDR transporter transcripts [33].

The miRNAs identified in our study play crucial roles in cancer cell biology. Using the miRDB target prediction program [39], *NKIRAS1*, which is a NFKB inhibitor was identified as one of the targets of *miR-1308*. Nuclear factor kappa B (NFκB) is a family of transcription factors that play important roles in regulating cell differentiation, proliferation, immune response and blocking apoptosis [40,41]. This family of transcription factors have been reported to chemosensitize P-gp overexpressing cancer cells [42]. This miRNA has also been shown to be upregulated in cancerous tissues and also in the more aggressive inflammatory breast cancer (IBC) in comparison to the non-IBC tissues [43,44]. Similarly, *miR-1246* targets the NKF3 kinase family member gene, *SGK269* (miRDB database). NKF3 or *PEAK1* promotes anchorage independent growth and tumour progression in pancreatic cancer cells transplanted in mice [45]. miR-149* is known to induce apoptosis by the direct inhibition of *Akt1* and *E2F1* in neuroblastoma cells [46]. *Akt* is the key kinase of the signal pathway, which mediates the regulation of divergent cellular processes including apoptosis, proliferation, differentiation and metabolism [47].

*miR-638* has been found to be consistently, highly expressed in human plasma and its presence in the plasma may be physiologically necessary [48]. As such, the ratio of *miR-92a/miR-638* in blood is associated with diagnosis in acute leukaemia patients [48]. Gene targets of *miR-638*, cyclin G2 and transcription elongation regulator 1-like factor (miRDB database), were involved in p53 and platelet-derived growth factor (PDGF) signalling pathways [49]. *miR-638* was one of the downregulated miRNAs in colorectal liver metastases with respect to the adjacent liver tissues that have the potential to serve as a prognostic and predictive marker of colorectal liver metastases [50]. Likewise, miR-1228* has been previously shown to be highly expressed in malignant mesothelioma tumour samples compared to normal samples [51].

The role of the miRNAs and their targeted pathways in the cells examined in this study are currently unknown. It is feasible that the same miRNAs may serve similar functions in these cells as is the case in the other reported cancers. However, the pathway analysis of the predicted targets of the 7 identified miRNAs in this study showed the maximum percentage of target genes to be significantly related to “**pathways in cancer**” and at least seven other pathways as well that were cancer related (Figure 8). The malignancy-related upregulated expression of these miRNAs may serve as potential biomarker in the treatment of cancer.

Chemotherapy comprises the major therapeutic strategy for clinical cancer treatment. However, chemotherapy fails to eliminate all tumour cells because of intrinsic or acquired drug resistance, which is the most common cause of tumour recurrence [52,53]. The role of miRNAs in the regulation of resistance mediated by multidrug transporters has only been examined recently [33]. Interestingly, we found that some of the significantly expressed miRNAs (like *miR-455-3p*) identified in this study target the multidrug resistant protein, P-glycoprotein (P-gp). For example, miRDB target prediction shows MDR member 1 or (P-gp) and *HIF1AN* (hypoxia-inducible factor 1, alpha subunit inhibitor) as *miR-455-3p* targets. HIF-1 alpha has been shown to induce MDR in hepatocellular carcinoma cell line [54]. In this study, the microarray analysis showed that the resistant leukaemia cell line has a lower expression level of this miRNA relative to its drug sensitive cells, which is consistent with its overexpression of P-gp. In the acquired cells we observed a suppression of *miR-455-3p* implicating potentially increased P-gp levels after MP transfer. Hence, our previous observations of P-gp protein transfer in the drug sensitive recipient cells after MP coculture [32] may be due to the transfer of these regulatory miRNAs together with protein via the MP cargo. In breast cancer, the overexpression of *miR-923* was shown to be upregulated in the Taxol resistant cancer cells relative to the normal cells [55].

Other than pathways related to malignancies and MP vesiculation, “**ABC transporters**” was identified as a significant biological pathway with the highest percentages of the identified miRNA target genes. Previous studies have also reported on the role of miRNAs involved in MDR in cancer. These include *miR-27a* and *miR-451,* whose expressions were shown to induce *MDR1*/P-gp expression in resistant human ovarian cancer cells [56]. The overexpression of *MRP1* and *miR-326* levels was inversely related in breast cancer tissues and leukaemia [33,57]. Recently, *miR-345* and *miR-7* have shown to target *MRP1* in MDR breast cancer cells relative to parental cells [58]. Apart from MDR, the ABC transporter, *ABCA1* has also been shown to possess *floppase* activity and is related to MP production [59]. In addition, of the significant predicted pathways identified for the miRNAs in this study, two of them namely: “**calcium signalling pathway**” and “**regulation of actin cytoskeleton**” were related to MP vesiculation (Figure 8). This suggests that MPs not only carry the enzymes for its release but also carry miRNAs and genes which may be potentially involved in its production and release.

Our study gives an implication of the role miRNAs contained within the MP cargo may play in contributing to the emergence of MDR and in regulating transporter expression in recipient cancer cells. Previous studies have shown miRNA transferred by other vesicular bodies like microvesicles to be functional in the recipient cells [60,61]. However, the functional role of the identified miRNAs in this study needs further exploration to have any clinical relevance.

## **Conclusions**

In conclusion, this study reveals miRNAs not only as oncogenic or tumour suppressive, but also highlights the potential role of these molecules as a potential class of diagnostic biomarkers across both haematological and non-haematological malignancies. The miRNAs transferred by MPs also play an important role in the regulation of biological processes involved in anticancer drug resistance. Indeed, the detection of circulating tumour-derived transcripts from melanoma, breast and lung cancer patients has identified MPs as potential markers of diagnostic and prognostic significance [26]. Thus, miRNA profiling has the potential to serve as a non-invasive approach to probe for the presence of deleterious cancer traits clinically.

## **Methods**

### **Cell Lines**

Two cell lines were used for these studies. The first cell line included the drug-sensitive human acute lymphoblastic leukaemia cell line CCRF-CEM (designated CEM for simplicity), and its MDR variant VLB_100_. The second included the drug-sensitive human breast adenocarcinoma cell line MCF-7, and its MDR variant MCF-7/DX (designated as DX for simplicity). These cells were kind gifts from Dr Rosanna Supino (Istituto Nazionale per lo Studio e la Cura dei Tumouri, Milan, Italy) and Dr Suzanne M. Cutts (La Trobe University, Victoria, Australia). Both of these cell lines have been validated earlier by our group as an appropriate model for the study of P-gp-mediated MDR *in vitro*[32,62,63]. All cell lines were cultured in RPMI 1640 (Invitrogen Australia, VIC, Australia) containing 10% FCS (Invitrogen, Australia) and maintained under a humidified incubator at 37°C in an atmosphere of 5% CO_2_.

### **MP harvesting and identification**

MPs were isolated from confluent CEM, VLB_100_ and DX cells by differential centrifugation, as previously described [32,33]. The MPs were designated as CEMMP, VLBMP and DXMP for simplicity. Briefly, culture supernatants were collected and centrifuged at 500 g for 5 min to pellet whole cells. The collected supernatant was re-centrifuged at 15,000 g for 1 h at 15°C to pellet the MPs. The final pellet was resuspended in serum free RPMI 1640 media and centrifuged at 2000 g for 1 min to remove debris. The clear MP suspension was further centrifuged at 18,000 g for 30 min at 15°C to pellet MPs. Validation of the isolated MP pellet was performed using flow cytometric analysis (FCM) (Cytomics FC500 MPL, Beckman Coulter) after FITC-annexin V (Beckman Coulter, NSW, Australia) staining as previously described [32]. Total protein content of MPs was determined using the Quant-iT™ protein assay as per the manufacturer’s instructions (Invitrogen Australia).

### **MP transfer experiment and isolation of mRNA**

In a 96-well U bottom culture plates, 180 μg of VLBMP or DXMP was cocultured with 1 x 10^5^ CEM or MCF-7 cells, respectively, for 4 h in total of 200 μL complete RPMI culture medium at 37°C and 5% CO_2_. Unbound MPs were removed by washing with PBS and centrifuging at 500 g for 5 min at 25°C after 4 h. The cocultured samples were designated as CEM + VLBMP and MCF-7+DXMP for simplicity and were referred to as the “acquired” cells.

Total RNA was extracted and pooled using Trizol® Reagent (Molecular Research Center, Inc, OH, U.S.A.) as per manufacturer’s recommendations from (i) the parental drug sensitive CEM or MCF-7 cells, (ii) the MDR strain VLB_100_ or DX cells, (iii) VLBMP or DXMP, and (iv) the cocultured samples CEM + VLBMP or MCF-7 + DXMP from duplicate experiments.

### **Gene expression analysis of MP vesiculation and miRNA biogenesis enzymes**

Quantitative real-time polymerase chain reaction (qRT-PCR) was used to assess the presence of s*cramblase* and *floppase* RNA transcripts (involved in MP budding), as well as *Argonaute, Dicer* and *Drosha* RNA transcripts (involved in miRNA biogenesis) in isolated MPs. Briefly, cDNA was synthesized using the Advantage RT-for-PCR Kit (Clontech Laboratories, Inc., Mountain View, CA). The specific primers against the target genes were used with GAPDH as the housekeeping primer (Sigma-Aldrich, St Louis, MO, USA) (Table 2). Reactions were carried out at the volume of 10 μL using 2 × SYBR Green Premix ExTaq (Takara Bio Inc., Shiga, Japan) with 10 pmole of target specific primer pairs and the amplification were performed on the LightCycler 2.0 (Roche, NSW, Australia). The thermal profile for the qRT-PCR was 95°C for 5 min followed by 45 cycles of 95°C for 5 sec, 55°C for 10 sec, and 72°C for 15 sec. The *Ct* data of each sample was compared with the housekeeping gene to obtain the Δ*C*t using the following formula: *ΔC*t = target gene *C*t − housekeeping gene *C*t. The relative expression level was calculated using *ΔΔC*t = 2^-*ΔC*t^ and expressed as fold difference from the experimental control ([*ΔΔC*t of sample ÷ *ΔΔC*t of drug-sensitive cells] x 1) as arbitrary units (a.u.).

**Table 2:** The list of sequences of primers used for real-time RT-PCR experiments

**Primers**	**Sequences**
*Scramblase*	Forward: 5'-AATGATTGGTGCCTGTTTCC -3' Reverse: 5'-TCCACTACCACACTCCTGATTT -3'
*Floppase*	Forward: 5'-TTGAACTAGGCAGCATCAGC-3' Reverse: 5'-GAACAGTGTCAACAGGCCAAT-3'
*Argonaute*	Forward: 5’-TTCATCGTGGTGCAGAAGAG-3’ Reverse: 5’-CCCAGAGGTATGGCTTCCTT-3’
*Dicer*	Forward: 5’-TCGCTGGCTGTAAAGTACGA-3’ Reverse: 5’-TTCAAGCAATTCTCGCACAG-3’
*Drosha*	Forward: 5’-TGCAACTGGTAGCCACAGAG-3’ Reverse: 5’-ACACTGCTGAAGCTGGGATT-3’
GAPDH	Forward: : 5’-TGCCAAATATGATGACATCAAGAA-3’ Reverse: 5’-GGAGTGGGTGTCGCTGTTG-3’
*miR-U6*	Forward: 5’-CTCGCTTCGGCAGCACA-3’ Reverse: 5’-AACGCTTCACGAATTTGCGT-3’
*miR-107*	5'-AGCAGCATTGTACAGGGCTATC-3'
*miR-125b*	5'-TCCCTGAGACCCTAACTTGTGA-3'
*miR-150*	5'-TCTTCCCAACCCTTGTACCAGTG-3'
*miR-210*	5'-CTGTGCGTGTGACAGCGGCTGA-3'

### **Analysis of RNA integrity and Affymetrix miRNA Arrays**

Total RNA integrity was analysed using the Agilent RNA 6000 Nano kit (Agilent Technologies Inc., Santa Clara, CA) and the result was analysed by Agilent 2100 Bioanalyzer (Agilent Technologies Inc.) as per the manufacturer’s recommendations; the RNA integrity number (RIN) of 10 represents the highest RNA integrity with minimal degradation and score of 1 is the lowest integrity [64]. Nanodrop-1000 spectrophotometer (Nanodrop technologies, DE, USA) was used for the quantification of RNA and 500 ng of RNA from each sample was used for miRNA microarray analysis. RNA labelling, hybridization (Affymetrix™ Fluidics Station 450), scanning (GeneChip® Scanner 3000 7 G) and raw data acquisition of the Affymetrix GeneChip ® miRNA Array (P/N 901326) were performed by Australian Genome Research Facility Ltd, VIC, Australia following a standard procedure from Affymetrix™ (Santa Clara,CA).

### **miRNA Microarray analysis**

#### ***Data processing***

Affymetrix “CEL” and “CHP” data files of each sample were processed with Affymetrix^TM^ miRNA QCTool software and following the guided workflow as described in the user manual (http://www.affymetrix.com/support/technical/manuals.affx). Briefly, the signal intensities data was extracted from data files and probes level intensity data were obtained using Wilcoxon-Rank Sum test, followed by background adjustment based on the GC content of ‘anti-genomic’ probes, quantile normalization, addition of a small constant (value 16) to avoid negative signal after background-GC correction, and finally applying median summarization to all probe set in each sample. Probe intensities data presented are all log_2_ transformed and *p*-values are obtained from the software after Wilcoxon-Rank Sum test. All microarray data discussed in this manuscript have been deposited in NCBI's Gene Expression Omnibus and are accessible through GEO Series accession number SEGSE34560 (http://www.ncbi.nlm.nih.gov/geo/query/acc.cgi?token=dvkxzwmqugw msrw&acc=GSE34560).

#### ***Data mining***

Data was filtered to include only annotated as *Homo sapiens* and miRNA, and probes with the *p*-value less than 0.06 (*p* < 0.06) were selected as a significant for further analysis. To identify those miRNAs that were correlated to drug resistance and its transfer from the donor to recipient cells by MPs, expression profiles of (i) MP donor cells (VLB_100_ or DX), (ii) isolated MPs (VLBMP or DXMP), and (iii) MP cocultured with drug sensitive cells (CEM + VLBMP or MCF-7+DXMP) were compared, and miRNAs with fold change more than 1.5 (FC > 1.5) were identified.

#### ***Hierarchical clustering and targeting pathway analysis***

‘Cluster 3.0’ program [65] was used for hierarchical clustering analysis, where selected miRNAs were clustered by centroid linkage using Euclidean distance, and depicted result was generated using ‘Java Tree View’ program [66]. Furthermore, a complete list of predicted gene targets on the selected miRNAs was downloaded from miRBase (Release version 16) (http://www.mirbase.org), miRDB ((http://www.mirdb.org) and the EMBL Nucleotide Sequence Database (http://www.ebi.ac.uk/embl/). The target genes for individual miRNAs with a score > 60 (miRBase) or with *p-*value < 0.01 (EMBL) were selected and uploaded to the online DAVID Bioinformatics Resources 6.7 program (http://david.abcc.ncifcrf.gov/) for their functional annotation clustering analysis. The biological pathways and gene regulation by the selected miRNAs were identified.

#### ***Microarray gene expression validation by qRT-PCR***

Total RNA from isolated MPs, acquired cells and whole cells were extracted as described above. cDNA for miRNA was synthesized using the NCode miRNA First Strand cDNA Module kit (Invitrogen Australia) on the GeneAmp PCRSystem 9700 (Applied Biosystems). *miR-150, miR-210, miR-107 and miR-125b* miRNA specific primers (10 pmole/reaction) were used for PCR for the detection of miRNAs using *miR-U6* as the housekeeping primer (all primers were from Sigma-Aldrich) (Table 2). SYBR Green qRT-PCR amplifications were performed on the Mastercycler® ep realplex (Eppendorf, NY, USA). Reactions were carried out in a 20 μL volume containing 10 μL of 2 × SYBR Green Premix ExTaq (Takara). The thermal profile for the qRT-PCR was 91°C for 5 min followed by 45 cycles of 91°C for 15 sec, 60°C for 30 sec, followed by melting curve detection. The *C*t data of each sample was collected automatically and data expressed as described above.

### **Statistical analysis**

A one-way analysis of variance (ANOVA) was used for comparison and statistical analysis between the sample populations and the control drug-sensitive cell population using the Graph Pad Prism software. *p*-values less than 0.05 were accepted as statistically significant.

## **Competing interests**

The authors declare that they have no competing interests.

## **Authors’ contributions**

RJ conducted all experiments and drafted the manuscript; FL participated in manuscript preparation including microarray and RT-PCR data analysis; JG participated in microarray breast cancer sample preparation; J-MM participated in the design of the microarray study and provided reagents; MB, GERG participated in work planning and manuscript preparation. All authors read and approved the final manuscript.

## References

[B1] Gong J Jaiswal R Mathys JM Combes V Grau GE Bebawy M Microparticles and their emerging role in cancer multidrug resistance Cancer Treat Rev 2011 12 12 10.1016/j.ctrv.2011.06.005 21757296

[B2] Morel O Morel N Jesel L Freyssinet J-M Toti F Microparticles: a critical component in the nexus between inflammation, immunity, and thrombosis Semin Immunopathol 2011 33 5 469 486 10.1007/s00281-010-0239-3 21866419

[B3] Coltel N Combes V Wassmer SC Chimini G Grau GE Cell vesiculation and immunopathology: implications in cerebral malaria Microbes Infect 2006 8 8 2305 2316 10.1016/j.micinf.2006.04.006 16829152

[B4] Janowska-Wieczorek A Marquez-Curtis Leah A Wysoczynski M Ratajczak Mariusz Z Enhancing effect of platelet-derived microvesicles on the invasive potential of breast cancer cells Transfusion (Paris) 2006 46 7 1199 1209 10.1111/j.1537-2995.2006.00871.x 16836568

[B5] van Doormaal FF Kleinjan A Di Nisio M Büller HR Nieuwland R Cell-derived microvesicles and cancer The Netherlands Journal of Medicine 2009 67 7 266 273 19687520

[B6] Burnier L Fontana P Kwak BR Angelillo-Scherrer A Cell-derived microparticles in haemostasis and vascular medicine Thromb Haemost 2009 101 3 439 451 19277403

[B7] Clark MR Flippin' lipids Nat Immunol 2011 12 5 373 375 10.1038/ni.2024 21502987

[B8] Hugel B Martínez MC Kunzelmann C Freyssinet J-M Membrane Microparticles: Two Sides of the Coin Physiology 2005 20 1 22 27 10.1152/physiol.00029.2004 15653836

[B9] Bassé F Gaffet P Bienvenüe A Correlation between inhibition of cytoskeleton proteolysis and anti-vesiculation effect of calpeptin during A23187-induced activation of human platelets: are vesicles shed by filopod fragmentation? Biochim Biophys Acta 1994 1190 2 217 224 10.1016/0005-2736(94)90077-9 8142419

[B10] Skog J Wurdinger T van Rijn S Meijer DH Gainche L Curry WT Carter BS Krichevsky AM Breakefield XO Glioblastoma microvesicles transport RNA and proteins that promote tumour growth and provide diagnostic biomarkers Nat Cell Biol 2008 10 12 1470 1476 10.1038/ncb1800 19011622 PMC3423894

[B11] Reich Iii CF Pisetsky DS The content of DNA and RNA in microparticles released by Jurkat and HL-60 cells undergoing in vitro apoptosis Exp Cell Res 2009 315 5 760 768 10.1016/j.yexcr.2008.12.014 19146850

[B12] Valadi H Ekstrom K Bossios A Sjostrand M Lee JJ Lotvall JO Exosome-mediated transfer of mRNAs and microRNAs is a novel mechanism of genetic exchange between cells Nat Cell Biol 2007 9 6 654 659 10.1038/ncb1596 17486113

[B13] Schetter AJ Harris CC Plasma microRNAs: a potential biomarker for colorectal cancer? Gut 2009 58 10 1318 1319 10.1136/gut.2009.176875 19749133

[B14] Lee YM Cho H-J Lee SY Yun SC Kim JH Lee SY Kwon SJ Choi E Na MJ Kang J-K Son JW MicroRNA-23a: A Novel Serum Based Diagnostic Biomarker for Lung Adenocarcinoma Tuberculosis and Respiratory Disease 2011 71 1 8 14 10.4046/trd.2011.71.1.8

[B15] Ambros V MicroRNA Pathways in Flies and Worms: Growth, Death, Fat, Stress, and Timing Cell 2003 113 6 673 676 10.1016/S0092-8674(03)00428-8 12809598

[B16] J-i S Tabunoki H Comprehensive analysis of human microRNA target networks BioData Min 2011 4 1 17 10.1186/1756-0381-4-17 21682903 PMC3130707

[B17] Asaga S Kuo C Nguyen T Terpenning M Giuliano AE Hoon DSB Direct Serum Assay for MicroRNA-21 Concentrations in Early and Advanced Breast Cancer Clin Chem 2011 57 1 84 91 10.1373/clinchem.2010.151845 21036945

[B18] Chen G-Q Zhao Z-W Zhou H-Y Liu Y-J Yang H-J Systematic analysis of microRNA involved in resistance of the MCF-7 human breast cancer cell to doxorubicin Med Oncol 2010 27 2 406 415 10.1007/s12032-009-9225-9 19412672

[B19] Croce CM Causes and consequences of microRNA dysregulation in cancer Nat Rev Genet 2009 10 10 704 714 10.1038/nrg2634 19763153 PMC3467096

[B20] Gonzales PA Pisitkun T Hoffert JD Tchapyjnikov D Star RA Kleta R Wang NS Knepper MA Large-scale proteomics and phosphoproteomics of urinary exosomes J Am Soc Nephrol 2009 20 2 363 379 10.1681/ASN.2008040406 19056867 PMC2637050

[B21] Landi MT Zhao Y Rotunno M Koshiol J Liu H Bergen AW Rubagotti M Goldstein AM Linnoila I Marincola FM Tucker MA Bertazzi PA Pesatori AC Caporaso NE McShane LM Wang E MicroRNA Expression Differentiates Histology and Predicts Survival of Lung Cancer Clin Cancer Res 2010 16 2 430 441 10.1158/1078-0432.CCR-09-1736 20068076 PMC3163170

[B22] Cimmino A Calin GA Fabbri M Iorio MV Ferracin M Shimizu M Wojcik SE Aqeilan RI Zupo S Dono M Rassenti L Alder H Volinia S Liu C-g Kipps TJ Negrini M Croce CM miR-15 and miR-16 induce apoptosis by targeting BCL2 Proc Natl Acad Sci U S A 2005 102 39 13944 13949 10.1073/pnas.0506654102 16166262 PMC1236577

[B23] Takeshita F Patrawala L Osaki M Takahashi R-u Yamamoto Y Kosaka N Kawamata M Kelnar K Bader AG Brown D Ochiya T Systemic Delivery of Synthetic MicroRNA-16 Inhibits the Growth of Metastatic Prostate Tumors via Downregulation of Multiple Cell-cycle Genes Mol Ther 2009 18 1 181 187 19738602 10.1038/mt.2009.207 PMC2839211

[B24] Taylor DD Gercel-Taylor C MicroRNA signatures of tumor-derived exosomes as diagnostic biomarkers of ovarian cancer Gynecol Oncol 2008 110 1 13 21 10.1016/j.ygyno.2008.04.033 18589210

[B25] Hunter MP Ismail N Zhang X Aguda BD Lee EJ Yu L Xiao T Schafer J Lee M-LT Schmittgen TD Nana-Sinkam SP Jarjoura D Marsh CB Detection of microRNA Expression in Human Peripheral Blood Microvesicles PLoS One 2008 3 11 e3694 10.1371/journal.pone.0003694 19002258 PMC2577891

[B26] El-Hefnawy T Raja S Kelly L Bigbee WL Kirkwood JM Luketich JD Godfrey TE Characterization of Amplifiable, Circulating RNA in Plasma and Its Potential as a Tool for Cancer Diagnostics Clin Chem 2004 50 3 564 573 10.1373/clinchem.2003.028506 14718398

[B27] Iero M Valenti R Huber V Filipazzi P Parmiani G Fais S Rivoltini L Tumour-released exosomes and their implications in cancer immunity Cell Death Differ 2007 15 1 80 88 17932500 10.1038/sj.cdd.4402237

[B28] Jung T Castellana D Klingbeil P Cuesta Hernandez I Vitacolonna M Orlicky DJ Roffler SR Brodt P Zoller M CD44v6 dependence of premetastatic niche preparation by exosomes Neoplasia 2009 11 10 1093 1105 19794968 10.1593/neo.09822 PMC2745675

[B29] Huber V Fais S Iero M Lugini L Canese P Squarcina P Zaccheddu A Colone M Arancia G Gentile M Seregni E Valenti R Ballabio G Belli F Leo E Parmiani G Rivoltini L Human Colorectal Cancer Cells Induce T-Cell Death Through Release of Proapoptotic Microvesicles: Role in Immune Escape Gastroenterology 2005 128 7 1796 1804 10.1053/j.gastro.2005.03.045 15940614

[B30] Al-Nedawi K Meehan B Rak J Microvesicles: Messengers and mediators of tumor progression Cell Cycle 2009 8 13 2014 2018 10.4161/cc.8.13.8988 19535896

[B31] Bussolati B Deregibus MC Camussi G Characterization of molecular and functional alterations of tumor endothelial cells to design anti-angiogenic strategies Curr Vasc Pharmacol 2010 8 2 220 232 10.2174/157016110790887036 19485921

[B32] Bebawy M Combes V Lee E Jaiswal R Gong J Bonhoure A Grau GER Membrane microparticles mediate transfer of P-glycoprotein to drug sensitive cancer cells Leukemia 2009 23 9 1643 1649 10.1038/leu.2009.76 19369960

[B33] Jaiswal R Gong J Sambasivam S Combes V Mathys JM Davey R Grau GE Bebawy M Microparticle-associated nucleic acids mediate trait dominance in cancer FASEB J 2011 30 30 10.1096/fj.11-186817 21965597

[B34] Daleke DL Phospholipid flippases J Biol Chem 2007 282 2 821 825 17130120 10.1074/jbc.R600035200

[B35] Pisitkun T Shen RF Knepper MA Identification and proteomic profiling of exosomes in human urine Proc Natl Acad Sci U S A 2004 101 36 13368 13373 10.1073/pnas.0403453101 15326289 PMC516573

[B36] Pegtel DM Cosmopoulos K Thorley-Lawson DA van Eijndhoven MA Hopmans ES Lindenberg JL de Gruijl TD Wurdinger T Middeldorp JM Functional delivery of viral miRNAs via exosomes Proc Natl Acad Sci U S A 2010 107 14 6328 6333 10.1073/pnas.0914843107 20304794 PMC2851954

[B37] Gibbings DJ Ciaudo C Erhardt M Voinnet O Multivesicular bodies associate with components of miRNA effector complexes and modulate miRNA activity Nat Cell Biol 2009 11 9 1143 1149 10.1038/ncb1929 19684575

[B38] Pigati L Yaddanapudi SCS Iyengar R Kim D-J Hearn SA Danforth D Hastings ML Duelli DM Selective Release of MicroRNA Species from Normal and Malignant Mammary Epithelial Cells PLoS One 2010 5 10 e13515 10.1371/journal.pone.0013515 20976003 PMC2958125

[B39] Wang X miRDB: a microRNA target prediction and functional annotation database with a wiki interface RNA 2008 14 6 1012 1017 10.1261/rna.965408 18426918 PMC2390791

[B40] Hayden MS Ghosh S Signaling to NF-κB Genes Dev 2004 18 18 2195 2224 10.1101/gad.1228704 15371334

[B41] Schmitz ML Mattioli I Buss H Kracht M NF-κB: A Multifaceted Transcription Factor Regulated at Several Levels ChemBioChem 2004 5 10 1348 1358 10.1002/cbic.200400144 15457532

[B42] Suttana W Mankhetkorn S Poompimon W Palagani A Zhokhov S Gerlo S Haegeman G Berghe W Differential chemosensitization of P-glycoprotein overexpressing K562/Adr cells by withaferin A and Siamois polyphenols Mol Cancer 2010 9 1 99 10.1186/1476-4598-9-99 20438634 PMC2873443

[B43] Wu Q Lu Z Li H Lu J Guo L Ge Q Next-generation sequencing of microRNAs for breast cancer detection J Biomed Biotechnol 2011 597145 26 10.1155/2011/597145 PMC3118289 21716661

[B44] Lerebours GC F Tozlu-Kara S Vacher S Lidereau R Bieche I MicroRNA Expression Profiling of Inflammatory Breast Cancer Thirty-Second Annual CTRC-AACR San Antonio Breast Cancer Symposium 2009 Cancer Research 2009: Abstract nr 6118, San Antonio, TX December 15, 2009

[B45] Wang Y Kelber JA Cao HST Cantin GT Lin R Wang W Kaushal S Bristow JM Edgington TS Hoffman RM Bouvet M Yates JR Klemke RL Pseudopodium-enriched atypical kinase 1 regulates the cytoskeleton and cancer progression Proc Natl Acad Sci 2010 107 24 10920 10925 10.1073/pnas.0914776107 20534451 PMC2890752

[B46] Lin R-J Lin Y-C Yu AL miR-149* induces apoptosis by inhibiting Akt1 and E2F1 in human cancer cells Mol Carcinog 2010 49 8 719 727 20623644 10.1002/mc.20647

[B47] Franke TF PI3K/Akt: getting it right matters Oncogene 2008 27 50 6473 6488 10.1038/onc.2008.313 18955974

[B48] Tanaka M Oikawa K Takanashi M Kudo M Ohyashiki J Ohyashiki K Kuroda M Down-Regulation of miR-92 in Human Plasma Is a Novel Marker for Acute Leukemia Patients PLoS One 2009 4 5 e5532 10.1371/journal.pone.0005532 19440243 PMC2678255

[B49] Dave RS Khalili K Morphine treatment of human monocyte-derived macrophages induces differential miRNA and protein expression: Impact on inflammation and oxidative stress in the central nervous system J Cell Biochem 2010 110 4 834 845 10.1002/jcb.22592 20564181 PMC2923828

[B50] Kahlert C Klupp F Brand K Lasitschka F Diederichs S Kirchberg J Rahbari N Dutta S Bork U Fritzmann J Reissfelder C Koch M Weitz J Invasion front-specific expression and prognostic significance of microRNA in colorectal liver metastases Cancer Sci 2011 102 10 1799 1807 10.1111/j.1349-7006.2011.02023.x 21722265

[B51] Guled M Lahti L Lindholm PM Salmenkivi K Bagwan I Nicholson AG Knuutila S CDKN2A, NF2, and JUN are dysregulated among other genes by miRNAs in malignant mesothelioma—A miRNA microarray analysis Genes Chromosomes Cancer 2009 48 7 615 623 10.1002/gcc.20669 19396864

[B52] Broxterman HJ Gotink KJ Verheul HMW Understanding the causes of multidrug resistance in cancer: a comparison of doxorubicin and sunitinib Drug resistance updates : reviews and commentaries in antimicrobial and anticancer chemotherapy 2009 12 4 114 126 19648052 10.1016/j.drup.2009.07.001

[B53] Fojo T Multiple paths to a drug resistance phenotype: Mutations, translocations, deletions and amplification of coding genes or promoter regions, epigenetic changes and microRNAs Drug resistance updates : reviews and commentaries in antimicrobial and anticancer chemotherapy 2007 10 1 59 67 17350322 10.1016/j.drup.2007.02.002

[B54] Zhu H Chen XP Luo SF Guan J Zhang WG Zhang BX Involment of hypoxia-inducible factor-1-alpha in multidrug resistance induced by hypoxia in HepG2 cells J Exp Clin Cancer Res 2005 24 4 565 574 16471319

[B55] Zhou M Liu Z Zhao Y Ding Y Liu H Xi Y Xiong W Li G Lu J Fodstad O Riker AI Tan M MicroRNA-125b Confers the Resistance of Breast Cancer Cells to Paclitaxel through Suppression of Pro-apoptotic Bcl-2 Antagonist Killer 1 (Bak1) Expression J Biol Chem 2010 285 28 21496 21507 10.1074/jbc.M109.083337 20460378 PMC2898411

[B56] Zhu H Wu H Liu X Evans BR Medina DJ Liu C-G Yang J-M Role of MicroRNA miR-27a and miR-451 in the regulation of MDR1/P-glycoprotein expression in human cancer cells Biochem Pharmacol 2008 76 5 582 588 10.1016/j.bcp.2008.06.007 18619946 PMC2628586

[B57] Liang Z Wu H Xia J Li Y Zhang Y Huang K Wagar N Yoon Y Cho HT Scala S Shim H Involvement of miR-326 in chemotherapy resistance of breast cancer through modulating expression of multidrug resistance-associated protein 1 Biochem Pharmacol 2010 79 6 817 824 10.1016/j.bcp.2009.10.017 19883630

[B58] Pogribny IP Filkowski JN Tryndyak VP Golubov A Shpyleva SI Kovalchuk O Alterations of microRNAs and their targets are associated with acquired resistance of MCF-7 breast cancer cells to cisplatin Int J Cancer 2010 127 8 1785 1794 10.1002/ijc.25191 20099276

[B59] Combes V Coltel N Alibert M van Eck M Raymond C Juhan-Vague I Grau GE Chimini G ABCA1 gene deletion protects against cerebral malaria: potential pathogenic role of microparticles in neuropathology Am J Pathol 2005 166 1 295 302 10.1016/S0002-9440(10)62253-5 15632021 PMC1602289

[B60] Kosaka N Ochiya T Unraveling the Mystery of Cancer by Secretory microRNA: Horizontal microRNA Transfer between Living Cells Front Genet 2011 2 97 3 22303391 10.3389/fgene.2011.00097 PMC3262223

[B61] Yang M Chen J Su F Yu B Lin L Liu Y Huang JD Song E Microvesicles secreted by macrophages shuttle invasion-potentiating microRNAs into breast cancer cells Mol Cancer 2011 10 117 21939504 10.1186/1476-4598-10-117 PMC3190352

[B62] Bebawy M Morris MB Roufogalis BD Selective modulation of P-glycoprotein-mediated drug resistance Br J Cancer 2001 85 12 1998 2003 10.1054/bjoc.2001.2184 11747345 PMC2364021

[B63] Dönmez Y Akhmetova L İşeri Ö Kars M Gündüz U Effect of MDR modulators verapamil and promethazine on gene expression levels ofMDR1andMRP1in doxorubicin-resistant MCF-7 cells Cancer Chemotherapy and Pharmacology 2011 67 4 823 828 10.1007/s00280-010-1385-y 20563580

[B64] Schroeder A Mueller O Stocker S Salowsky R Leiber M Gassmann M Lightfoot S Menzel W Granzow M Ragg T The RIN: an RNA integrity number for assigning integrity values to RNA measurements BMC Mol Biol 2006 7 3 10.1186/1471-2199-7-3 16448564 PMC1413964

[B65] de Hoon MJL Imoto S Nolan J Miyano S Open source clustering software Bioinformatics 2004 20 9 1453 1454 10.1093/bioinformatics/bth078 14871861

[B66] Saldanha AJ Java Treeview–extensible visualization of microarray data Bioinformatics 2004 20 17 3246 3248 10.1093/bioinformatics/bth349 15180930

